# ABCC8 mRNA expression is an independent prognostic factor for glioma and can predict chemosensitivity

**DOI:** 10.1038/s41598-020-69676-7

**Published:** 2020-07-29

**Authors:** Kaijia Zhou, Yanwei Liu, Zheng Zhao, Yinyuan Wang, Lijie Huang, Ruichao Chai, Guanzhang Li, Tao Jiang

**Affiliations:** 1 Beijing Neurosurgical Institute, Capital Medical University, Beijing, 100070 China; 2 Department of Neurosurgery, Beijing Tiantan Hospital, Capital Medical University, Beijing, 100070 China; 3 Center of Brain Tumor, Beijing Institute for Brain Disorders, Beijing, 100070 China; 4 China National Clinical Research Center for Neurological Diseases, Beijing, 100070 China

**Keywords:** Biomarkers, Medical research, Molecular medicine, Neurology

## Abstract

Glioma is the most common primary intracranial tumor and is associated with very low survival rates. The development of reliable biomarkers can help to elucidate the molecular mechanisms involved in glioma development. Here the expression of ABCC8 mRNA, clinical characteristics, and survival information based on 1893 glioma samples from four independent databases were analyzed. The expression patterns of ABCC8 mRNA were compared by a Chi square test. The overall survival rate of gliomas was evaluated according to the expression level of ABCC8 mRNA. The prognostic value of this marker in gliomas was tested using Cox single factor and multi factor regression analyses. We found patients with low WHO grade, oligodendrocytoma, low molecular grade, IDH mutation, and 1p19q combined deletion had high ABCC8 mRNA expression. The patients with high expression of ABCC8 mRNA had longer survival. ABCC8 mRNA expression was a new independent prognostic index for glioma. Temozolomide chemotherapy was an independent index to prolong overall survival in high ABCC8 mRNA expression glioma patients, whereas in patients with low expression, there was no significant difference. So ABCC8 mRNA expression could be an independent prognostic indicator for glioma patients and could predict the sensitivity of glioma to temozolomide.

## Introduction

Glioma is the most common primary intracranial tumor, accounting for 81% of malignant brain tumors^1^. World Health Organization (WHO) grade 4 glioblastoma is the most malignant form of glioma with a 5-year relative survival rate of 5%^2^. In recent years, studies have elucidated some genetic changes in glioma, such as IDH1/2^3^, TP53^4^ and ATRX^5^ mutations, TERT promoter mutations^6^, MET-exon-14-skipping (METex14), PTPRZ1-MET (ZM) fusions, MET amplification^7^, MGMT promoter methylation^8^, and 1p/19q co-deletion^9^, which have been helpful in guiding the classification and treatment of glioma. As per the WHO’s classification of central nervous system tumors in 2016, for the first time, diffuse gliomas were classified according to IDH1 or IDH2 mutations and the co-deletion of 1p and 19q chromosome arms^10^. However, the development of new and reliable biomarkers is necessary to further elucidate the molecular mechanism of glioma development.

ATP-binding cassette (ABC) transporters play a crucial role in the development of resistance by the efflux of anticancer agents outside of cancer cells. It was found that the expression of ABCC8 was down-regulated in pancreatic cancer^11^, lung adenocarcinoma^12^ and triple negative breast cancer^13^. The low expression of ABCC8 was associated with poor prognosis in these tumors. Thompson et al.^14^. found ABCC8 expression was greater in supratentorial ependymoma compared to glioblastoma and metastases. However the expression and clinical significance of ABCC8 mRNA in gliomas are still unclear.

In this study, we collected the expression data and clinical information with respect to ABCC8 mRNA based on 656 glioma samples from CGGA database as testing set. The expression patterns of ABCC8 in different types of gliomas were compared and the overall survival (OS) rate of glioma patients was evaluated according to the expression level of ABCC8 mRNA, and the prognostic value of this marker in gliomas was tested. Meanwhile the OS evaluated according to the expression level of ABCC8 mRNA was validated by the other three publicly available sets as GSE16011 (n = 264), REMBRANDT (n = 348) and TCGA (n = 625).

## Results

### Characteristics of patients in this study

Gene expression data and the clinical data were collected from the CGGA and organized. Clinical characteristics included age, gender, histopathology, WHO classification, IDH mutation status, 1p/19q col-deletion status, radiotherapy, chemotherapy, WHO classification (2016) and overall survival. The patients were divided into two groups according to the median age of 42 years (Table 1).

**Table 1:** Relationship between the clinical features of glioma and ABCC8 mRNA expression in CGGA. *According to the median age of 42 years, the patients were divided into two groups. LGG: lower grade glioma (WHO II and WHO III). GBM: glioblastoma.

Parameter	Variables	N	ABCC8 mRNA expression				χ^2^	P value
			Low	%	High	%		
Age*	< 42	303	148	48.80	155	51.20	0.301	0.584
	≥ 42	353	180	51.00	173	49.00		
Gender	Female	282	137	48.60	145	51.40	0.398	0.528
	Male	374	191	51.10	183	48.90		
Histopathology	Oligodendroglioma	144	28	19.40	116	80.60	115.567	0.000
	Astrocytoma	274	122	44.50	152	55.50		
	Glioblastoma	238	178	74.80	60	25.20		
WHO grade	II	176	49	27.80	127	72.20	99.684	0.000
	III	242	101	41.70	141	58.30		
	IV	238	178	74.80	60	25.20		
IDH mutation	Wildtype	277	197	71.10	80	28.90	67.345	0.000
	Mutant	331	125	37.80	206	62.20		
	NA	48						
1p/19q codel	Non-codel	461	251	54.40	210	45.60	60.950	0.000
	Codel	141	24	17.00	117	83.00		
	NA	54						
Radiotherapy	No	134	50	37.30	84	62.70	10.023	0.002
	Yes	495	261	52.70	234	47.30		
	NA	27						
Chemotherapy	No	157	60	38.20	97	61.80	11.314	0.001
	Yes	471	253	53.70	218	46.30		
	NA	28						
WHO grade (2016)	IDH Mutant, 1p/19q Codel (LGG)	114	21	18.40	93	81.60	114.393	0.000
	IDH Mutant, 1p/19q Non-codel (LGG)	171	75	43.9	96	56.1		
	IDH Wildtype (LGG)	95	52	54.7	43	45.3		
	IDH Mutant (GBM)	46	29	63.0	17	37.0		
	IDH wildtype (GBM)	182	145	79.7	37	20.3		
	NA	48						

### Correlations between ABCC8 mRNA expression and clinical features with glioma

After dividing the patients into low/high ABCC8 mRNA expression groups according to the median value of ABCC8 mRNA expression, the relationships between clinical features and ABCC8 mRNA expression were analyzed. Histopathology (p = 0.000), WHO grade (p = 0.000), IDH mutation (p = 0.000), 1p/19q codel (p = 0.000), radiotherapy (p = 0.002), chemotherapy (p = 0.001) and WHO grade (2016) (p = 0.000) were significantly correlated with ABCC8 mRNA expression. ABCC8 mRNA expression was higher in patients with better prognosis indicators, such as oligodendroglioma, low WHO grade, IDH mutation, 1p/19q col-deletion and low WHO molecular grade (2016) (Table 1).

### High ABCC8 mRNA expression in patients with better prognosis indicators

**Figure 1:**
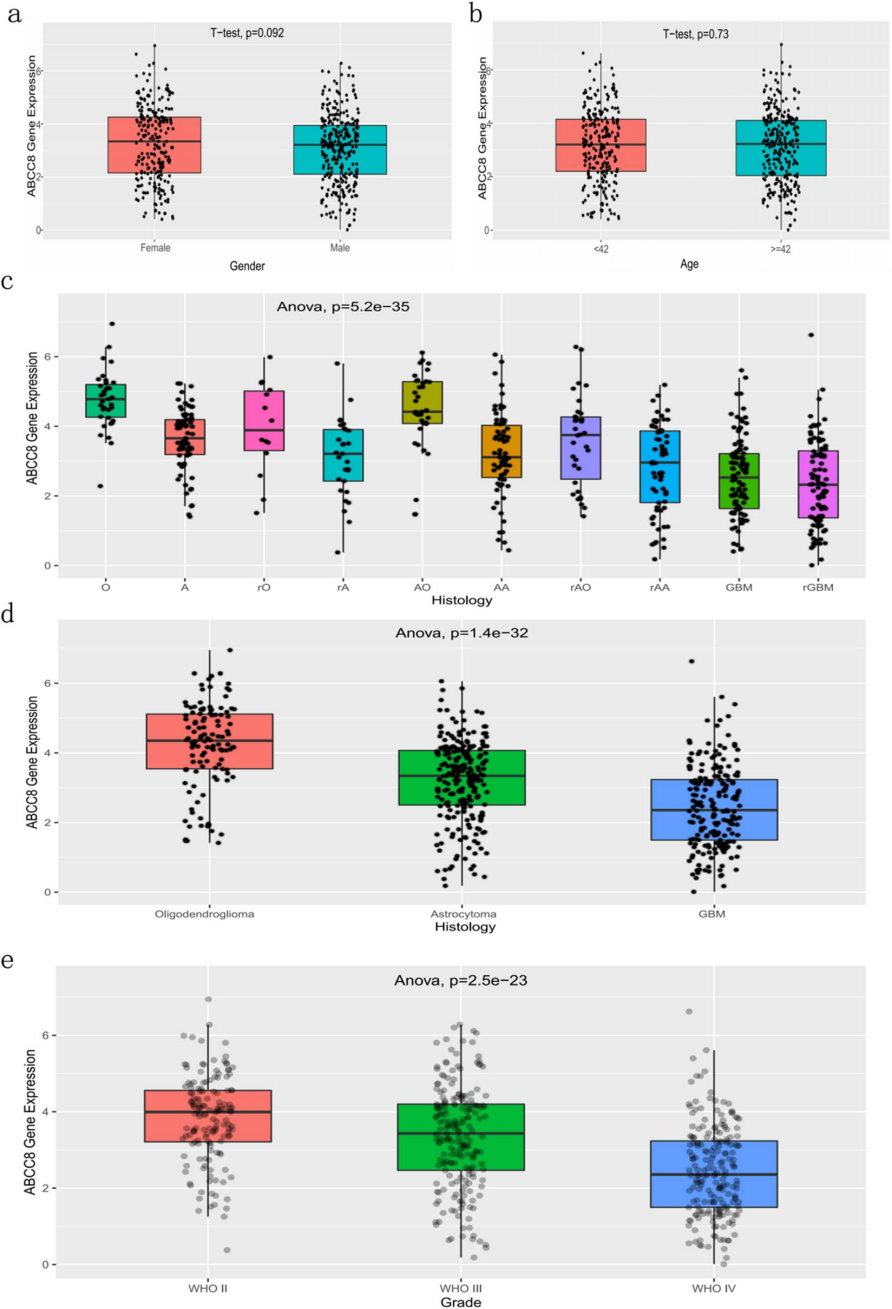
Analysis of ABCC8 mRNA expression according to gender, age, histopathology, and WHO grade in CGGA. Comparison of gender (**a**), age (**b**), histology (**c**,**e**), and WHO grade (**f**). n = 656 per group. Data represent mean ± SD. In (**a**,**b**), p by Student’s t-test. In (**c**–**e**), p by one-way ANOVA test.

**Figure 2:**
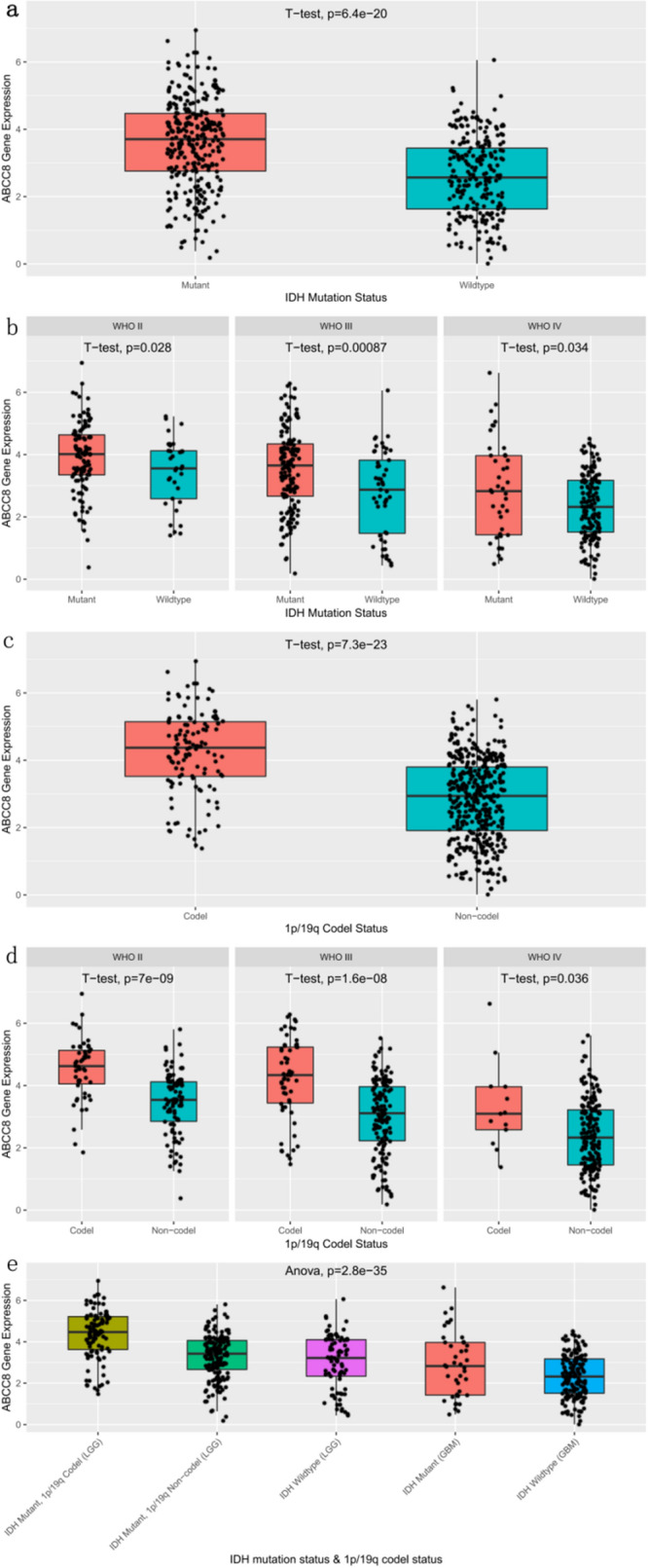
Analysis of ABCC8 mRNA expression according to IDH mutation and 1p/19q col-deletion status. Subgroup analysis of ABCC8 mRNA expression according to WHO classification in CGGA. Different IDH mutation status (**a**) and 1p/19q deletion status (**c**) were compared. (**f**) Comparison of WHO molecular classification of glioma in 2016. In (**a**), n = 608. In (**c**), n = 602. In (**b**,**d**), n (II) = 176, n (III) = 242, n (IV) = 238. Data represent mean ± SD. p by Student’s t-test.

The ABCC8 mRNA expression was compared via box plots, among diferent clinical and molecular pathological characteristics, including gender, age, histopathology, WHO grade (Fig. 1), IDH mutation and 1p/19q col-deletion status (Fig. 2). The patients were divided into two groups according to the median age of 42 years. The results showed that ABCC8 mRNA expression was higher in patients with better prognosis indicators, such as oligodendroglioma (p = 1.4e−32), low WHO grade (p = 2.5e−23), IDH mutation (p = 6.4e−20), 1p/19q col-deletion (p = 7.3e−23) and low who molecular grade (2016) (p = 2.8e−35).

### High ABCC8 mRNA expression predicts good prognosis of OS

**Figure 3:**
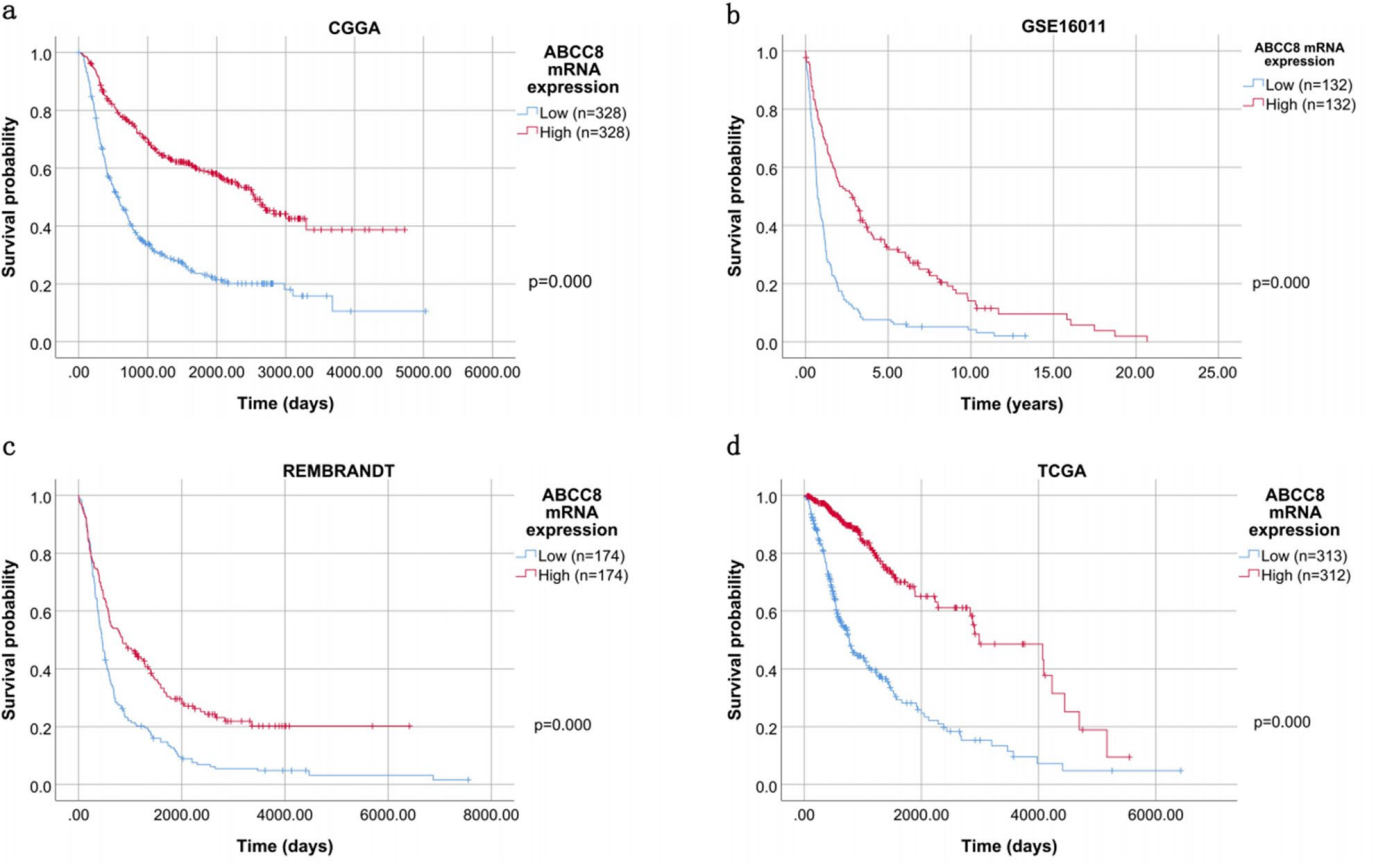
Kaplan–Meier curves for OS according to ABCC8 mRNA expression in CGGA. Verifcation in the GSE16011 database, REMBRANDT and TCGA database. Kaplan–Meier curves for OS in CGGA (**a**), GSE16011 (**b**), REMBRANDT (**c**), and TCGA database (**d**). p by Log Rank (Mantel-Cox) test.

Kaplan–Meier survival curves were constructed to explore the prognostic value of ABCC8 mRNA expression in OS. The results showed that high expression of ABCC8 mRNA was related to better OS than low expression (P = 0.000; Fig. 3), which was also confirmed by using the GSE16011 (p = 0.000), REMBRANDT (p = 0.000) and TCGA databases (p = 0.000) (Fig. 3).

### Subgroup analysis revealed the prognostic value of ABCC8 mRNA expression in terms of OS

**Figure 4:**
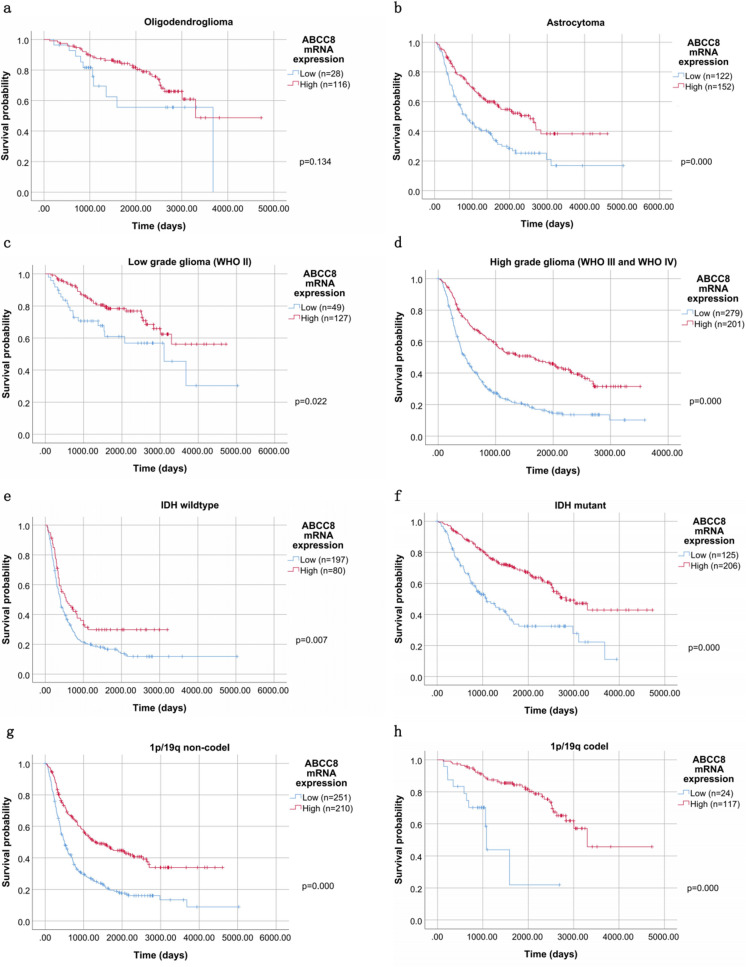
Kaplan–Meier curves for OS according to ABCC8 mRNA expression in CGGA. Subgroup analysis of OS was performed based on Kaplan–Meier curves according to histologic type, WHO classification, IDH mutation and 1p/19q col-deletion status. Kaplan–Meier curves for OS according to histologic type (**a**,**b**), WHO classification (**c**,**d**), IDH mutation (**e**,**f**) and 1p/19q col-deletion status (**g**,**h**). p by Log Rank (Mantel-Cox) test.

Subgroup analysis revealed that the high expression of ABCC8 mRNA was correlated with good OS in astrocytoma (p = 0.000), low WHO grade (p = 0.022), high WHO grade (p = 0.000), IDH mutant (p = 0.000), IDH wildtype (p = 0.007), 1p/19q codel (p = 0.000), 1p/19q non codel glioma patients (p = 0.000) (Fig. 4).

### ABCC8 mRNA expression is an independent predictor of OS

Univariate Cox analysis identifed the potential OS-related variables, including the age, gender, histopathology, WHO classification, IDH mutation status, 1p/19q col-deletion status, radiotherapy, chemotherapy and ABCC8 mRNA expression. Multivariate Cox analyses showed that high ABCC8 mRNA expression (HR value: 0.704, 95% CI 0.543–0.913, P = 0.008), IDH mutation status (HR value: 0.750, 95% CI 0.569–0.989, p = 0.041), 1p/19q col-deletion status (HR value: 0.425, 95% CI 0.265–0.682, p = 0.000) and chemotherapy (HR value: 0.676, 95% CI 0.499–0.914, p = 0.011) were independent predictors of long OS. While age (HR value: 1.499, 95% CI 1.181–1.903, p = 0.001), WHO classification (HR value: 2.312, 95% CI 1.807–2.958, p = 0.000) were independent predictors of poor OS (Table 2).

**Table 2:** Univariate and multivariate analysis of the correlation of ABCC8 mRNA expression with OS among glioma patients in CGGA. CI = confidence interval; HR = hazard ratio.

Variable	Univariate cox regression			Multivariate cox regression		
	p value	HR	95% CI (lower–upper)	p value	HR	95% CI (lower–upper)
Gender	0.357	1.100	0.898–1.346			
Age	0.000	1.690	1.378–2.072	0.001	1.499	1.181–1.903
Histopathology	0.000	2.122	1.874–2.403	0.834	0.978	0.794–1.204
WHO grade	0.000	2.697	2.328–3.124	0.000	2.312	1.807–2.958
IDH mutant	0.000	0.318	0.258–0.392	0.041	0.750	0.569–0.989
1p/19q codel	0.000	0.264	0.190–0.366	0.000	0.425	0.265–0.682
Radiotherapy	0.080	1.266	0.973–1.648	0.063	0.746	0.548–1.016
Chemotherapy	0.084	1.241	0.972–1.584	0.011	0.676	0.499–0.914
ABCC8 mRNA expression	0.000	0.376	0.306–0.463	0.008	0.704	0.543–0.913

### High ABCC8 mRNA expression predicts chemosensitivity

Afer dividing the patients into low/high ABCC8 mRNA expression groups according to the median value of ABCC8 mRNA expression, Univariate Cox analysis identifed the potential OS-related variables, including the age, gender, histopathology, WHO grade, IDH mutation status, 1p/19q col-deletion status, radiotherapy and chemotherapy. Multivariate Cox analyses showed that in high ABCC8 mRNA expression group, chemotherapy (HR value: 0.526, 95% CI 0.326–0.848, p = 0.008) and IDH mutation status (HR value: 0.427, 95% CI 0.274–0.664, p = 0.000) were independent predictors of long OS. While age (HR value: 1.991, 95% CI 1.329–2.983, p = 0.001), WHO grade (HR value: 2.429, 95% CI 1.735–3.402, p = 0.000) were independent predictors of poor OS (Table 3). In low ABCC8 mRNA expression group, there was no significant difference between the survival of patients treated with temozolomide and those not treated with the drug (Univariate Cox:p = 0.867, Multivariate Cox:p = 0.085) (Table 4). These results showed that high ABCC8 mRNA expression could predict the sensitivity of glioma to temozolomide.

**Table 3:** Univariate and multivariate analysis of the correlation of radiotherapy and chemotherapy with OS among high ABCC8 mRNA expression glioma patients in CGGA. CI = confidence interval; HR = hazard ratio.

Variable	Univariate cox regression			Multivariate cox regression		
	p value	HR	95% CI (lower–upper)	p value	HR	95% CI (lower–upper)
Gender	0.014	1.533	1.092–2.152	0.161	1.320	0.895–1.946
Age	0.000	2.023	1.438–2.845	0.001	1.991	1.329–2.983
Histopathology	0.000	1.975	1.654–2.359	0.645	1.072	0.798–1.438
WHO grade	0.000	2.898	2.269–3.701	0.000	2.429	1.735–3.402
IDH mutant	0.000	0.294	0.206–0.420	0.000	0.427	0.274–0.664
1p/19q codel	0.000	0.322	0.216–0.481	0.179	0.640	0.334–1.226
Radiotherapy	0.195	1.299	0.875–1.928	0.809	1.059	0.664–1.691
Chemotherapy	0.533	1.125	0.777–1.628	0.008	0.526	0.326–0.848

**Table 4:** Univariate and multivariate analysis of the correlation of radiotherapy and chemotherapy with OS among low ABCC8 mRNA expression glioma patients in CGGA. CI = confidence interval; HR = hazard ratio.

Variable	Univariate cox regression			Multivariate cox regression		
	p value	HR	95% CI (lower–upper)	p value	HR	95% CI (lower–upper)
Gender	0.212	0.851	0.661–1.097			
Age	0.001	1.562	1.209–2.018	0.098	1.292	0.953–1.752
Histopathology	0.000	1.874	1.547–2.270	0.608	0.921	0.672–1.262
WHO grade	0.000	2.044	1.681–2.486	0.000	2.316	1.584–3.388
IDH mutant	0.000	0.465	0.353–0.613	0.516	0.889	0.623–1.268
1p/19q codel	0.006	0.412	0.218–0.779	0.012	0.349	0.153–0.792
Radiotherapy	0.439	0.869	0.608–1.241	0.014	0.608	0.409–0.905
Chemotherapy	0.867	1.029	0.740–1.430	0.085	0.709	0.479–1.049

### Low ABCC8 mRNA expression predicts radiosensitivity

Multivariate Cox analyses showed that in low ABCC8 mRNA expression group, radiotherapy (HR value: 0.608, 95% CI 0.409–0.905, p = 0.014) and 1p/19q codel status (HR value: 0.349, 95% CI 0.153–0.792, p = 0.012) were independent predictors of long OS. While WHO grade (HR value: 2.316, 95% CI 1.584–3.388, p = 0.000) were independent predictors of poor OS (Table 4). In high ABCC8 mRNA expression group, there was no significant difference between the survival of patients treated with radiotherapy and those not treated with radiotherapy (Univariate Cox:p = 0.195, Multivariate Cox:p = 0.809) (Table 3). These results showed that low ABCC8 mRNA expression could predict the sensitivity of glioma to radiotherapy.

## Discussion

ABCC8 (ATP binding cassette subfamily C member 8), also known as ABC36 (member 36 of ATP binding cassette transporter superfamily), SUR1 (sulfonylurea receptor 1), or MRP8, is encoded by the ABCC8 gene located at 11p15.1. Gene Ontology (GO) notes related to the ABCC8 gene include potassium channel activity, ATP binding, sulfonylurea receptor activity, ATPase activity (GO molecular function for the ABCC8 gene). The ABCC8 gene encodes proteins that are members of the ATP binding cassette (ABC) transporter superfamily. ABC proteins transport various molecules (based on the Entrez gene summary for ABCC8 gene). The research findings of Martin et al.^15^ show that the protein encoded by ABCC8 plays a role in regulating the ATP-sensitive potassium (K-ATP) channel and insulin release. The ATP sensitive potassium channel is a protein complex that can couple the cell energy level with cell excitability and control a wide range of physiological processes, including hormone secretion, neuronal transmission, vasodilation, and cardiac and neuron pretreatment for ischemic injury. Studies of Zhang et al.^16^ show that K-ATP channels are widely expressed in the central nervous system and are coupled with cell metabolism and electrical activity. The K-ATP channel in mature substantia nigra dopaminergic neurons consists of inward rectifying K-channel subunit 6.2 and SUR1, which regulates the K-ATP channel in a Parkinson’s disease mouse model and has protective effects on dopamine neurons. ABCC8-related diseases include diabetes, permanent neonatal and hyperinsulinemic hypoglycemia, and familial disease 1. The related pathways include inward rectifying K + channel and energy metabolism integration. Mutations and deletions in the protein (genecards summary for ABCC8 gene) were observed in infants with hyperinsulinemic hypoglycemia.

ABCC8 is a transmembrane protein that regulates the activity of ion channels in neurons, glia, and endothelial cells. In neuroinflammatory diseases, SUR1 expression was found to be upregulated. In rodent models of subarachnoid hemorrhage^17^, ischemic stroke^18^, traumatic brain injury^19^, spinal cord injury^20^, and brain metastasis^21^, the inhibitory effect of glibenclamide on SUR1 was found to reduce edema and neuroinflammation by antagonizing cytotoxic edema and apoptosis. A retrospective study carried out to examine patients with diabetic acute ischemic stroke led to the finding that glibenclamide could improve neurological functions, reduce the conversion of bleeding, and reduce mortality^22,23^. The preclinical study of Thompson et al.^21^, using a brain metastasis mouse model, illustrated the potential benefits of glibenclamide for the prevention of edema. JHA Ruchira et al.^24^ showed that SUR1 and its related transient receptor potential cation channel subfamily-m (Trpm4) channel are the key factors of brain edema and intracranial hypertension in traumatic brain injury and other neurological diseases. Glibenclamide reduces brain edema and intracranial hypertension by inhibiting this channel. The SNP encoding SUR1 can be used as a predictor of increases in intracranial pressure. Further, King et al.^25^ demonstrated that sur1-trpm4 is a key target of stroke. After ischemia, the sur1-trpm4 channel is upregulated in all cells of the neurovascular unit (including neurons, astrocytes, microglia, oligodendrocytes, and microvascular endothelial cells). Moreover, the second-generation sulfonylurea glibenclamide can inhibit SUR1 at nanomolar concentration to reduce the brain swelling and death of patients.

At present, the expression and function of ABCC8 mRNA in brain tumors is less studied. Thompson et al.^14^ analyzed six glioblastomas, 12 brain metastases, 11 medulloblastomas, nine supratentorial ependymomas, and eight posterior fossa ependymomas by immunofluorescence. The expression of ABCC8 and its co-localization with blood vessels, neurons, and glial cells was analyzed and compared by ANOVA. The results showed that the percentage of cells expressing ABCC8 in total tissue area (mean ± SD) was 3.9 ± 4 in glioblastoma, 4.1 ± 3.1 in brain metastasis, 8.2 ± 7.2 in medulloblastoma, 9.1 ± 7 in supratentorial ependymoma, and 8.1 ± 5.9 in the posterior cranial cavity. The expression of ABCC8 was higher in supratentorial ependymoma than in glioblastoma and metastasis (P < 0.05), and higher in medulloblastoma than in glioblastoma. .It is suggested that ABCC8 expression is lower in malignant brain tumors. This is consistent with our findings.

Our study found that the expression of ABCC8 mRNA was negatively correlated with the tumor tissue type, WHO grade, WHO molecular grade, IDH wildtype, and 1p/19q non-codel. The patients with oligodendroglioma, low WHO grade, low WHO molecular grade, IDH mutation, and 1p19q deletion had high ABCC8 mRNA expression. This is associated with their good prognosis, and a long survival period..ABCC8 mRNA was found to be an independent factor associated with good prognosis. However, the molecular mechanism underlying such effects has not been reported and requires further study. Mohelnikova et al.^11^ found that the expression of ABCC8 in ductal adenocarcinoma of the pancreas was significantly downregulated compared to levels in adjacent non-tumor tissues. It was concluded that stem cells might play a role in the development and progression of ductal adenocarcinoma of the pancreas. Wang et al.^12^ screened 6 genes (CLEC17A, TAGAP, ABCC8, BCAN, FLT3, and CCR2) related to immune and stromal cells in the tumor microenvironment through bioinformatics and constructed a risk assessment model to predict the prognosis of lung adenocarcinoma. They found patients with high expressions of CLEC17A, TAGAP, ABCC8, FLT3, and CCR2 had better prognosis and higher OS within 5 years. They demonstrated through a series of rigorous analyses that lung adenocarcinoma patients with high infiltration of immune cells (stromal cells) had better prognosis and earlier staging. The study of Hlavá et al.^13^ showed that the expression level of ABCC8 in breast cancer is significantly correlated with the grade and expression of hormone receptors, which represents a potential modifying factor of chemotherapy progress and the response of breast cancer. ABCC8, C11, C12, C13, and A10 showed the highest level of downregulation within the cluster BC3, which significantly prevailed in ERnegative (p = 0.012), PRnegative (p = 0.009), and TNBC (p = 0.022) cases, that is, in patients with a generally worse prognosis. Zhou et al.^26^ performed quantitative analysis of ABCC8 mRNA expression in breast cancer cells using real-time RT-PCR. The ABCC8 mRNA expression level was found to be related to the methylation state of the CpG island in the promoter region,that is, the expression of mRNA was higher in the low methylation state of the promoter region. Thus, the potential application of CpG island methylation in the promoter region of breast cancer cells to predict the chemosensitivity of breast cancer was indicated. In this study, we found that temozolomide chemotherapy was an independent index to prolong the survival of glioma patients with high expression of ABCC8 mRNA, but there was no significant difference in the survival of patients with low expression. This sugested that high ABCC8 mRNA expression could predict chemosensitivity of glioma. Our study found that in low ABCC8 mRNA expression glioma patients, radiotherapy was independent predictors of long OS. While in high ABCC8 mRNA expression group, there was no significant difference. This sugested that low ABCC8 mRNA expression could predict radiosensitivity of glioma. The mechanism needs further study.

## Methods

### Clinical specimen collection

Glioma tissues, the corresponding genomic data and the patients' follow-up information (histopathology, gender, age, WHO grade, molecular pathological results, overall survival and censor status, etc.) were obtained from the Chinese Glioma Genome Atlas (CGGA, including patients treated at Beijing Tiantan Hospital, Sanbo Hospital in Beijing, Tianjin Medical University General Hospital, The First Affiliated Hospital of Nanjing Medical University, Harbin Medical University, China Medical University). All research performed was approved by the Tiantan Hospital Institutional Review Board (IRB) and kept consistent with the principles of the Helsinki Declaration. All the subjects were diagnosed with gliomas by consensus, according to central pathology reviews by independent board-certified neuropathologists and further graded based on the 2007/2016 WHO classification. Informed consent was obtained from all subjects or, if subjects are under 18, from a parent and/or legal guardian. All experimental methods were carried out in accordance with the relevant guidelines and regulations. The establishment and management of the CGGA database has been reported previously^27^. The specimens were collected under IRB KY2013-017-01 and were frozen in liquid nitrogen within 5 min of resection. Clinical information and RNA-sequencing expression results were collected from the GSE16011 dataset (https://www.ncbi.nlm.nih.gov/geo/), REMBRANDT dataset (https://caitegrator-info.nci.nih.gov/rembrandt) and TCGA dataset (https://cancer.nih.gov).

### mRNA sequencing data

#### mRNA sequencing

Before preparing the library, use RNeasy Mini Kit (Qiagen) to separate the total RNA according to the manufacturer's instructions. The 2,100 biological analyzer (Agilent Technologies) was used to check the RNA intensity, and only the high quality samples whose RNA Integrity Number value (RIN) was greater than or equal to 6.8 were used to construct the sequencing library. In addition to the superscript III reverse transcriptase (Invitrogen) for the synthesis of the first strand of cDNA, 1 μg total RNA is usually used in conjunction with the TruSeq RNA library preparation kit (Illumina). The concentration of the ligated fragment with the adapter was enriched and purified by PCR, and the DNA concentration with the adapter was determined by quantitative PCR (Applied Biosystems 7,500). The length of DNA fragment was measured by 2,100 biological analyzer, and the insertion size was 200 bp. Then, the RNA sequence library is sequenced using the Illumina HiSeq 2000/2,500/4,000 sequencing system. The library adopts paired end strategy, and the reading length is 101 bp, 125 bp or 150 bp respectively.

### Mapping and quantification

STAR (v2.5.2b Dobin et al., 2012) and RSEM (v1.2.31 Li et al. 2011) software were used to process the location and quantification of RNA sequences. In a nutshell, these readings are consistent with the Human Genome reference (GENCODE v19 Magi HG19), and then use RSEM to calculate the sequence read count for each GENCODE gene. The expression levels of different samples are combined into a FPKM (fragments per kilobase transcriptome per million fragments per million fragments) matrix. We define an expression gene only when the expression level of half of the samples is greater than 0. Last, we only retained the genes expressed in the mRNA expression profile.

### RNA sequence quantification

After mapping reads to the human genome (GENCODE v19 and HG19), we used the RSEM software (v1.2.31) to quantify the expression level of genes/transcripts from RNA sequence data. The preparation, sequencing and data analysis of the RNAseq library are the same as our previous studies^28^.

### Statistical analysis

According to the median value of ABCC8 mRNA expression, glioma patients were separated into low and high expression groups in the CGGA, GSE16011, REMBRANDT and TCGA databases. Box plots were used to evaluate the ABCC8 mRNA expression in the subgroups according to clinical and molecular characteristics such as gender, age, histopathology, WHO grade, IDH mutation, 1p/19q codel status, and WHO molecular grade (2016) by R sofware (version 3.6.1) (https://www.r-project.org/) and related packages. Chi-square tests were utilized to evaluate correlations between the expression of ABCC8 mRNA and clinical information by SPSS 25.0 software package. The OS was calculated from the date of histological diagnosis until death or the last follow-up. Kaplan–Meier survival analysis and log-rank tests were used to evaluate the statistical significance associated with stratified survival groups by SPSS 25.0 software package. The potential prognostic factors were selected by Cox analysis. Correlations between ABCC8 mRNA expression, survival and other clinical characteristics of glioma patients were confrmed by using multifactor Cox analysis. P values less than 0.05 were considered statistically significant.

## Conclusions

Our study preliminarily confirmed the expression of ABCC8 mRNA in glioma and its clinical significance. ABCC8 mRNA expression can be used as an independent prognostic indicator for glioma patients. Its high expression can be used to predict the chemosensitivity of glioma. Meanwhile low ABCC8 mRNA expression can be used to predict the sensitivity of glioma to radiotherapy. However, the molecular mechanism of its action needs to be elucidated by further studies.
